# Evidence of two lineages of the dengue vector *Aedes aegypti* in the Brazilian Amazon, based on mitochondrial DNA *ND4* gene sequences

**DOI:** 10.1590/S1415-47572009005000036

**Published:** 2009-03-27

**Authors:** Raimundo Sousa Lima, Vera Margarete Scarpassa

**Affiliations:** 1 Programa de Pós Graduação em Genética, Conservação e Biologia Evolutiva, Instituto Nacional de Pesquisas da Amazônia, Manaus, AMBrazil; 2 Laboratório de Genética de Populações e Evolução de Vetores de Malária e Dengue, Instituto Nacional de Pesquisas da Amazônia, Manaus, AMBrazil

**Keywords:** * Aedes aegypti*, dengue vector, genetic structure, gene flow, Brazilian Amazon, genetic lineages

## Abstract

Genetic variation was estimated in ten samples populations of *Aedes aegypti* from the Brazilian Amazon, by using a 380 bp fragment of the mitochocondrial NADH dehydrogenase subunit 4 (*ND4*) gene. A total of 123 individuals were analyzed, whereby 13 haplotypes were found. Mean genetic diversity was slightly high (*h* = 0.666 ± 0.029; π = 0.0115 ± 0.0010). Two AMOVA analyses indicated that most of the variation (~70%-72%) occurred within populations. The variation found among and between populations within the groups disclosed lower, but even so, highly significant values. *F*_ST_ values were not significant in most of the comparisons, except for the samples from Pacaraima and Rio Branco. The isolation by distance (IBD) model was not significant (*r* = 0.2880; p = 0.097) when the samples from Pacaraima and Rio Branco were excluded from the analyses, this indicating that genetic distance is not related to geographic distance. This result may be explained either by passive dispersal patterns (via human migrations and commercial exchange) or be due to the recent expansion of this mosquito in the Brazilian Amazon. Phylogenetic relationship analysis showed two genetically distinct groups (lineages) within the Brazilian Amazon, each sharing haplotypes with populations from West Africa and Asia.

## Introduction

*Aedes aegypti* is the main vector of urban yellow fever and four dengue virus serotypes (DENV-1 to DENV-4). This mosquito is also involved in the transmission of other arboviruses and filarial helminthes which affect humans and several other animal species (Forattini, 2002). However, the main epidemiological problem is related to the transmission of dengue, especially in its more severe form, dengue hemorrhagic fever (DHF) (Gubler, 1998). It is estimated that worldwide 50-100 million cases of dengue fever (DF) occur every year, with 500,000 cases of DHF and at least 22,000 deaths, mainly among children (WHO, 2007). Until now, no vaccine against dengue is available, the efforts to curb the progress of this disease being based solely on vector control measures alone (Gubler, 1998).

*Aedes aegypti* is an urban species, being well adapted to live in close association with humans and demonstrating great adaptive capacity to the most varied environments (Paupy *et al.*, 2000; Donalísio and Glasser, 2002). Studies have shown that ecological variation, human intervention, dispersal patterns and the constant use of insecticides may affect the genetic population structure of this vector (Bosio *et al.*, 1998; Yan *et al.*, 1998; Huber *et al.*, 2002; Paupy *et al.*, 2005; Scarpassa *et al.*, 2008), this having been associated with heterogeneous patterns of vector competence in the transmission of dengue and urban yellow fever viruses (Failloux *et al.*, 2002; Lourenço-de-Oliveira *et al.*, 2002, 2004).

In 1955, *Ae. aegypti* came to be considered as having been eradicated from Brazil (Consoli and Lourenço-de-Oliveira, 1994). Nevertheless, a few years later it was probably re-introduced into the country through the states of Pará (1967), Bahia (1976) and Rio de Janeiro (1977) (Lourenço-de-Oliveira *et al.*, 2004). In 1998, this vector was already present countrywide (Figueiredo, 2003; MS, 2007). In spite of vector control programs, dengue outbreaks are common, with a significant increase in cases of DHF (MS, 2007).

The Amazon region possesses ideal characteristics favoring rapid development of the life cycle, proliferation and longevity of *Ae. aegypti*, namely in the form of towns with large harbors, the tropical climate (high temperatures and humidity), pronounced rainy seasons and complex environmental and social factors. Currently, this vector is found in virtually all the towns of the Brazilian Amazon (SVS, 2006), its dispersion possibly having occurred via river traffic, a major travel route for persons and commercial trade between many localities of the region. Thus, studies in population genetics of these *Ae. aegypti* populations could provide knowledge on gene-flow patterns and colonization events, which would be important parameters for outlining new strategies for local and regional vector control.

Among the mitochondrial genes, the NADH dehydrogenase subunit 4 (*ND4*) gene has shown to be an excellent marker for analyzing the genetic population structure and colonization events in *Ae. aegypti* (Gorrochotegui-Escalante *et al.*, 2002; Bosio *et al.*, 2005; Costa-da-Silva *et al.*, 2005; Herrera *et al.*, 2006; Bracco *et al.*, 2007; Paduan and Ribolla, 2008; Urdaneta-Marquez *et al.*, 2008).

In this study we investigated the genetic variability of wild *Ae. aegypti* populations from seven towns of the Brazilian Amazon, through the use of sequences of the *ND4* gene. We also analyzed four suburban neighborhoods within the city of Manaus, in order to establish the gene flow pattern at the micro-geographic level.

**Figure 1:**
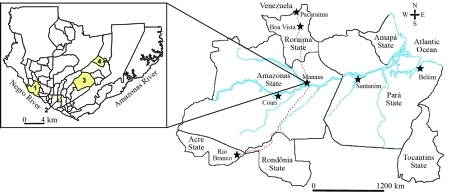
Collection sites of *Aedes aegypti* in the Brazilian Amazon. On the larger map, rivers are represented in blue, whereas highways are represented as red dashed lines. The stars indicate collection sites. On the smaller map, the four urban neighborhoods of Manaus city are represented in yellow: 1 = Compensa; 2 = Praça 14 de Janeiro; 3 = Coroado; 4 = Tancredo Neves.

**Figure 2:**
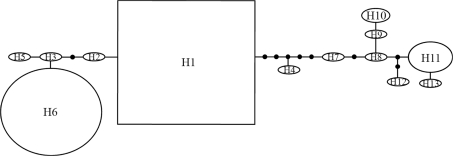
Haplotype network observed in the ten samples of *Aedes aegypti*. The size of the ellipse is proportional to the number of individuals found for each haplotype. Black circles indicate mutational steps between haplotypes.

**Figure 3:**
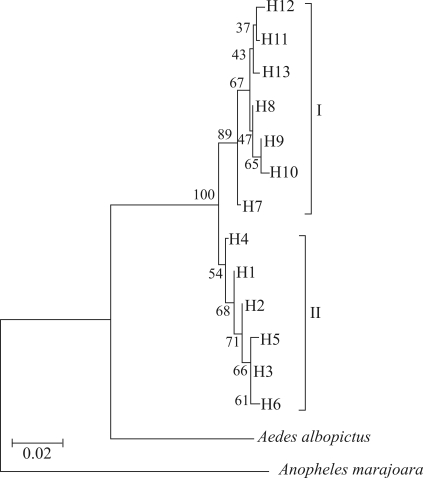
Phylogenetic relationships among *Aedes aegypti* haplotypes, based on the neighbor-joining (NJ) algorithm under the Tamura-Nei genetic distance model. Bootstrap values are marked on the branches.

## Materials and Methods

### Collection of the mosquito

*Ae. aegypti* samples were collected in seven towns of the Brazilian Amazon: Belém and Santarém (state of Pará), Boa Vista and Pacaraima (state of Roraima), Rio Branco (state of Acre), and Coari and Manaus (state of Amazonas). With a view to micro-geographic analysis, individuals from four suburban neighborhoods of Manaus were sampled: Coroado, Praça 14 de Janeiro, Compensa, and Tancredo Neves (Figure 1). Information on localities, states and geographic coordinates is presented in Table 1. A total of ten samples were analyzed.

Larval and pupal stages were collected from 15 to 30 diverse artificial recipients in each town, this including the four suburbs of Manaus. The specimens collected from each recipient-site were transfered to Laboratory of Population Genetics and Evolution of Malaria and Dengue Vectors in the National Institute for Amazonian Research, in Manaus, Amazonas, Brazil. Larvae and pupae were reared to adulthood in the laboratory. Subsequently, specimens were identified by means of a key developed by Forattini (2002), to be subsequently frozen at -80 °C until genomic DNA extraction. One to two individuals from each artificial recipient were used in this study.

### DNA extraction, and amplification and sequencing of the *ND4* gene

Genomic DNA was extracted individually from 4^th^ instar larvae and/or adults by using the protocol developed by Collins *et al.* (1987). A 380 bp fragment was amplified with the aid of primers described by Gorrochotegui-Escalante *et al.* (2002), under amplification conditions as outlined by Bosio *et al.* (2005). A negative control was used in all PCR reactions. PCR products were deposited on 1% agarose gels, stained with ethidium bromide and analyzed under UV light. These were then purified using GFX PCR DNA and a Gel Band Purification Kit (GE Healthcare, UK) according to manufacturer's recommendations. Subsequently, they were sequenced in an automated *MegaBACE 1000 Analysis System* sequencer (GE Healthcare, UK). All individuals were sequenced in both directions.

### Statistical analysis

Sequences were edited and aligned by means of the BioEdit (Hall, 1999) and Chromas (Griffith University, Queensland, Australia) programs. Haplotype genealogy based on the parsimony method was generated through the TCS program (Clement *et al.*, 2000). The genetic diversity of the haplotypes (*h*) and nucleotides (π), the number of variable sites (NV) and the average number of nucleotide differences (*K*), were calculated by using the DnaSP program (Rozas *et al.*, 2003). Tajima's *D* (Tajima, 1989) and Fu's *F*s (Fu, 1997) neutrality tests, as well as haplotype (*h*) and nucleotide (π) diversities, the average number of nucleotide differences (*K*) and sequence divergence (*D*) calculated between the two haplotype groups, were also obtained through the DnaSP program.

Estimates of genetic distance and gene flow based on *F*_ST_ and *Nm* values, respectively, as well as hierarchical analysis (AMOVA), were calculated using the Arlequin program (Excoffier *et al.*, 2006). *F*_ST_ statistics (Wright, 1921) was used to estimate the genetic structure of the populations. The number of migrants per generation (*Nm*), which provides an estimate of gene flow between sub-populations, was obtained through *F*_ST_ values. The correlation between genetic (*F*_ST_) and geographic (km) distances was estimated by using Mantel's test (Mantel, 1967). The isolation by distance (IBD) hypothesis was tested with the IBDWS program (Jensen *et al.*, 2005). Geographic distances for this analysis were obtained from the GPS and those in the Google Earth program. Levels of significance were adjusted by the Bonferroni correction whenever there were multiple tests (Rice, 1989).

Phylogenetic relationships among haplotypes were estimated by using the Mega program (Kumar *et al.*, 2004), based on the neighbor-joining (NJ) algorithm within the Tamura-Nei genetic distance model. Bootstrap support was calculated by means of 1000 replicates. In this analysis, *Aedes albopictus* (GenBank accession number: EF153761) and *Anopheles marajoara* (GenBank accession number: AY846347) were employed as outgroups.

The haplotypes in this study were compared with those available in previous inquiries, from Mexico - AF334841-AF334865 (Gorrochotegui-Escalante *et al.*, 2002), Peru - DQ177153-DQ177155 (Costa-da-Silva *et al.*, 2005), and the Americas, Africa and Asia - DQ176828-DQ176831, DQ176833-DQ176843 and DQ176845-DQ176849 (Bracco *et al.*, 2007). Only shared haplotypes are shown in Table 2. Those detected in Thailand (Bosio *et al.*, 2005) and Venezuela (Herrera *et al.*, 2006; Urdaneta-Marquez *et al.*, 2008) have not yet been deposited in the GenBank, thereby precluding comparison with haplotypes in this study. The haplotypes observed in Brazil by Paduan and Ribolla (2008) (AY906835-AY906853) were not compared with those of the present study, since there was no indication of origin. The haplotypes of this study are deposited in the GenBank under accession numbers EU650405-EU650417.

## Results

### Distribution and frequency of haplotypes

13 haplotypes were discovered from among 123 sequenced individuals of *Ae. aegypti* (Table 2), haplotype 1 being the most frequent (47.15%) followed by haplotype 6 (32.52%). Except for Boa Vista and Pacaraima, the latter was detected in all populations. Haplotype 11, the third most frequent haplotype, was observed in three suburbs of Manaus and in Boa Vista. Samples from Belém revealed the highest number of exclusive haplotypes (H3, H8, H9 and H10), among which H10 was observed in four individuals, whereas H3, H8 and H9 were detected in only one single individual each. Haplotype 1, represented by a rectangle, is probably the oldest (Clement *et al.*, 2000), being separated from H6 and H11 by three and nine mutational steps, respectively (Figure 2).

### Genetic variability and gene flow among the populations

The highest levels of both haplotype and nucleotide diversity were detected in the samples from Belém and two suburbs of Manaus (Coroado and Praça 14 de Janeiro) (Table 3). The samples from Tancredo Neves (in Manaus) displayed the highest nucleotide diversity, whereas there was no nucleotide variation in those from Pacaraima (H1) and Rio Branco (H6), thus indicating these to be monomorphic. Tajima's *D* and Fu's *F*s neutrality tests showed no significant results (p > 0.05) in all the samples, this suggesting that these populations are in genetic equilibrium (Fu, 1997).

Two analyses levels were used in the AMOVA test to verify the origin of genetic variation in the different hierarchical levels and groups. In the first, all the populations analyzed were considered as constituting a single group (island model), the highest variation occurring within the populations themselves (72.69%). Nevertheless, the percentage of variation was lower among samples (27.31%), even though highly significant (*F*_ST_ = 0.273; p < 10^-5^). In the second AMOVA test, the populations were grouped according to their respective states, the Amazonas group, comprising the four suburbs of Manaus (Coroado, Praça 14 de Janeiro, Compensa and Tancredo Neves) and Coari, the Pará group, comprising Santarém and Belém, the Roraima group with Boa Vista and Pacaraima, and the Acre group with Rio Branco. Similar to the first AMOVA analysis, the greater part of variation (70.66%) occurred within the populations themselves, with a highly significant value (*F*_ST_ = 0.293, p < 10^-5^). The percentage of variation between populations within the states (18.87%) was also highly significant (*F*_SC_ = 0.210, p < 10^-5^), whereas among the states, the percentage of variation (10.47%) was low and insignificant (*F*_CT_ = 0.104; p > 0.01) (data not shown).

Significant *F*_ST_ values (p < 0.001) were observed, after Bonferroni correction, in most of the comparisons involving samples from Rio Branco, with the corresponding *Nm* values varying from 0.0 to 1.0, thereby indicating from an absent to a reduced gene flow (Table 4). *F*_ST_ values were high in the six comparisons involving samples from Pacaraima, three of which becoming statistically significant after the Bonferroni correction. There were no statistically significant comparisons among samples from the four suburbs of Manaus, although a high *F*_ST_ value (0.366; *Nm* = 0.9) was obtained between samples from Compensa and Tancredo Neves, which was not significant after Bonferroni correction. This could be explained on considering that in Compensa only group II haplotypes were found, whereas in the samples from Tancredo Neves, haplotypes of two groups in similar frequencies were sampled.

The correlation between genetic and geographic distances was statistically significant (*r* = 0.5815; p = 0.006) when taking all the populations into consideration, thereby suggesting isolation by distance (IBD). However, when the samples from Pacaraima and Rio Branco, the most genetically structured, were removed from the analysis, correlation was non-significant (*r* = 0.2880; p = 0.097), thus indicating that gene flow is not related to geographic distance.

### Phylogenetic analysis

Phylogenetic relationships among the 13 haplotypes recovered two groups, with a bootstrap support of 89% between them (Figure 3). Group I was composed of seven haplotypes, the third most frequent (H11), H10 (from Belém) and five unique haplotypes (H7, H8, H9, H12 and H13). In this group, haplotype H7 observed in Santarém, was the most distant from the remainder. Furthermore, there were tree subgroups in group I: 1) cluster H11, H12 and H13, composed of samples from Manaus and Boa Vista; 2) cluster H8, H9 and H10, comprising the sample from Belém; and 3) H7, consisting of the sample from Santarém. However, bootstrap support values for splits leading to these groups were low. Group II consisted of six haplotypes, the two most common and widespread (H1 and H6) and four unique haplotypes (H2, H3, H4 and H5).

Haplotype diversity within groups I and II was 0.6571 and 0.5276, respectively, whereas nucleotide diversity was 0.00590 and 0.00325, respectively. The average number of nucleotide differences (*K*) between groups I and II was 10.308, whereas nucleotide divergence (*D*) was 0.02713.

## Discussion

### Distribution and frequency of haplotypes

In this study, although H1 manifested the highest frequencies in the samples from Pacaraima (100%), Boa Vista (83%) and Santarém (79%), it was totally absent in those from Tancredo Neves, Belém and Rio Branco. These findings suggest that gene flow is absent between Pacaraima and Rio Branco, and reduced between Pacaraima and Belém, and Boa Vista and Rio Branco (see Table 4). H1 was found by Costa-da-Silva *et al.* (2005) in Peru (as H3), and by Bracco *et al.* (2007) (as H15) in the localities of Boa Vista, Manaus, Cariacica (state of Espirito Santo), Santos (state of São Paulo), and Piura (Peru), but was not observed in Mexico (Gorrochotegui-Escalante *et al.*, 2002). Nevertheless, the haplotypes in this study were not compared with haplotypes from Venezuela since the latter were not available (Herrera *et al.*, 2006). Based on these results, we were unable to propose an origin for H1.

On the contrary, H6 was absent in the samples from Pacaraima and Boa Vista, but was detected in all the others in this study. In an extensive study on the Americas, Africa and Asia, Bracco *et al.* (2007) observed that H6 (as H17) was exclusive to Brazil, with a wide distribution. H6 has not been detected neither in Mexico (Gorrochotegui-Escalante *et al.*, 2002) nor Peru (Costa-da-Silva *et al.*, 2005). It is our opinion that H6 may either have arisen in Brazil or was introduced from other, as yet unstudied, regions of the world. Additional inquiries may clarify the origin of this haplotype. H11, observed in Boa Vista and three suburbs in Manaus, was also discovered in Mexico as H6 (Gorrochotegui-Escalante *et al.*, 2002), and in other locations in Brazil (the towns of Boa Vista and Potim), besides the U.S.A. and Senegal (West Africa) as H7 (Bracco *et al.*, 2007). This haplotype may have arisen in West Africa, to later spread to other regions in the world. Thus, H11 may represent a further introduction into Brazil. H3, observed in only one individual from Belém, was also found in Mexico as H5 (Gorrochotegui-Escalante *et al.*, 2002) and Asia as H13 (Bracco *et al.*, 2007). This haplotype may have been introduced into the Americas (including Brazil) from Asia. H8, H9 and H10, exclusive of Belém, have no shared with any other haplotype from previous studies. These haplotypes may represent recent mutations or introductions that have not yet spread.

### Genetic variability and gene flow among the populations

In this study, mean genetic diversity proved to be relatively high, whereas both Costa-da-Silva *et al.* (2005), on studying three populations from Peru, and Bosio *et al.* (2005), when analyzing 19 populations from Thailand, estimated a lower value (π = 0.0079) than that presented herein. The highest values of nucleotide diversity were found in populations from Mexico (π = 0.0143; Gorrrochotegui-Escalante *et al.*, 2002), Venezuela (π = 0.0187; Herrera *et al.*, 2006), Brazil (π = 0.0174; Paduan and Ribolla, 2008) and the Americas-Africa-Asia (π = 0.0199; Bracco *et al.*, 2007). Comparing our π values to those of Venezuela, a lower haplotype number (7) was observed in the latter, whereas π values were higher, this possibly indicating that the Venezuelan populations are older than those of the Brazilian Amazon (Kambhampati and Rai, 1991). However, these findings could be the outcome of mosquito control efforts, with the consequential loss of intermediate haplotypic lineages, thus resulting in high divergence among haplotypes.

The highest level of genetic diversity found in the samples from Belém and suburbs of Manaus (Coroado, Praça 14 de Janeiro and Tancredo Neves) may be coincident with demographic density being the highest in these two port-towns in north Brazil, besides their both being situated on the banks of two large rivers (Amazon and Negro), thereby being exposed to an intense flow of both persons and trade. Furthermore, the industrial center of Manaus is one of the largest in Brazil. These factors are likely to favor multiple introductions and/or the dispersal of *Ae. aegypti*, thus contributing to an increase in gene flow between populations, with the subsequent rise in genetic variation. The four exclusive haplotypes found in Belém may support this hypothesis. These findings could imply that, despite the constant use of insecticides for vector control, these *Ae. aegypti* populations may have a large effective population size.

The two AMOVA analyses indicated that a greater part of the variation occurred within populations (~70%-72%), which can probably be attributed to the presence of two genetically distinct sympatric haplotype lineages. The significant variation (27.31%) among populations (non-grouped) could be primarily due to the high genetic differentiation found between Pacaraima and Rio Branco, whereas the significant variation (18.87%) between populations within the states themselves could result mainly from the differentiation observed between Belém and Santarém (Pará group). This indicates that a significant genetic structure occurs in *Ae. aegypti* from the Brazilian Amazon. Similar results were found in Mexico (Gorrochotegui-Escalante *et al.*, 2002) and Venezuela (Herrera *et al.*, 2006).

In this study, the gene flow pattern was related to H1 and H6 frequencies. In spite of the great distance between Santarém and Boa Vista (~880 km), gene flow was extensive (*Nm* = 35.1) due to the high frequency of H1 in both populations. The free gene flow (*Nm* = 13.1) between Belém and Tancredo Neves, about 1,284 km apart, was directly related to H6 frequency. Nevertheless, this result may indicate distinct introduction events followed by the recent expansion of *Ae. aegypti* in the Brazilian Amazon (less than 25 years) rather than the current gene flow.

Of the ten samples analyzed herein, those from Rio Branco and Pacaraima were the most genetically structured and isolated by distance. This is mainly why genetic variation was absent in these samples, theses results possibly being consistent with two hypotheses: 1) the Rio Branco and Pacaraima *Ae. aegypti* populations were founded by few individuals (founder effect) and have maintained low effective population sizes; or 2) the sizes of these populations were reduced due to the constant use of insecticides, thereby generating repeated bottleneck effects and/or populations founded by few individuals through cycles of extinction and re-colonization. Both effects could imply the reduction of genetic variability through genetic drift (Hartl and Clark, 1997), thus possibly resulting in highly divergent populations.

All told, we observed higher rates of gene flow for populations of the Brazilian Amazon when compared to the two previous studies undertaken in Brazil. Ayres *et al.* (2003) analyzed the *Ae. aegypti* populations of five Brazilian states with RAPD markers, and found high levels of genetic differentiation and reduced gene flow at both the macro-geographic (*Nm* = 0.54) and micro-geographic (*Nm* = 0.69) levels. Paduan *et al.* (2006) also used RAPD markers in populations from six other Brazilian states and observed reduced gene flow among all of these (*Nm* = 0.65), even though from the same state (*Nm* = 0.83). Compared to the present study, these results suggest that levels of genetic variability and differentiation among Brazilian *Ae. aegypti* populations are relatively high. Nevertheless, most of the populations of the Brazilian Amazon analyzed here manifested higher gene flow, probably due to human migration and river or land trading movement, thereby favoring the dispersal of this vector in the region. Alternatively, this could indicate the recent expansion of *Ae. aegypti* throughout this area. The absence of isolation by distance, when the samples from Pacaraima and Rio Branco were removed from the analyses, supports these hypotheses. Identical results were found in samples from Mexico (Gorrochotegui-Escalante *et al.*, 2002), Venezuela (Herrera *et al.*, 2006) and Brazil (Scarpassa *et al.*, 2008). Furthermore, the active dispersal patterns (flight) of *Ae. aegypti* have been estimated as being between 100 and 800 m (Ordonez-Gonzalez *et al.*, 2001; Honorio *et al.*, 2003), this dispersal mechanism obviously not being a possible explanation for our results.

Two haplotype groups were indicated through phylogenetic relationship analysis, which is in accordance with previous studies (Gorrrochotegui-Escalante *et al.*, 2002; Bosio *et al.*, 2005; Herrera *et al.*, 2006; Bracco *et al.*, 2007; Paduan and Ribolla, 2008). Group I shared haplotypes with Senegal (West Africa), whereas group II shared haplotypes with Asia. Due to the appearance of similar results with the mtDNA *COI* gene in most of our samples (Scarpassa *et al.*, 2008), we believe that H1 from group II may have originated in East Africa. Even though haplotype and nucleotide diversity values in group I were higher than in group II, the latter is probably the more ancient, since it presents the oldest and most widespread haplotypes. Bracco *et al.* (2007) found slightly higher values for *K* (12.015) and *D* (0.03207) between the two haplotype groups than those disclosed in the present study. This could be related to the colonization history of these populations (Scarpassa *et al.*, 2008). These results are consistent with the presence of two genetic lineages within the Brazilian Amazon, these being sympatric in Manaus, Boa Vista and Belém. Based on previous findings (Mousson *et al.*, 2005; Bracco *et al.*, 2007) and those arising from this study, group II (the older lineage) may have persisted independent of eradication programs in the Americas, this including in Brazil.

Concluding, the data in this study indicate multiple introductions of *Ae. aegypti* into the Brazilian Amazon. Cluster analysis clearly showed two genetic lineages, each of which sharing haplotypes with West Africa and Asia, thereby suggesting that Brazilian Amazon populations probably originated from these regions. The existence of distinct lineages within the Brazilian Amazon could imply differences in the susceptibility for transmitting dengue and urban yellow fever viruses (Beerntsen *et al.*, 2000; Failloux *et al.*, 2002; Lourenço-de-Oliveira *et al.*, 2004; Urdaneta-Marquez *et al.*, 2008) and in responses to vector control programs.

## Figures and Tables

**Table 1:** Localization of the *Aedes aegypti* populations from the Brazilian Amazon.

State	Locality	Location code	Coordinates	Sample size
Amazonas	Coroado*	Cor	3° 05' S, 59° 58' W	19
Amazonas	Praça 14 de Janeiro*	Pra	3° 07' S, 60° 00' W	11
Amazonas	Compensa*	Com	3° 06' S, 60° 03' W	10
Amazonas	Tancredo Neves*	TaN	3° 03' S, 59° 56' W	10
Amazonas	Coari	Coa	4° 05' S, 63° 08' W	12
Pará	Santarém	Stm	2° 26' S, 54° 43' W	14
Pará	Belém	Blm	1° 25' S, 48° 27' W	11
Roraima	Boa Vista	BoV	2° 48' N, 60° 42' W	12
Roraima	Pacaraima	Pac	4° 25' N, 61° 08' W	12
Acre	Rio Branco	RBr	9° 58' S, 67° 49' W	12

*neighborhoods of Manaus.

**Table 2:** Haplotype frequencies in the ten *Aedes aegypti* samples*,* and haplotypes shared by this study and three previous studies that used the *ND4* gene.

Haplotypes	Localities										Total			
	Cor	Pra	Com	TaN	Coa	Stm	Blm	BoV	Pac	RBr		Mex^1^	Per^2^	AAA^3^
H1	7	3	7		8	11		10	12		58		H3	H15
H2					1						1			
H3							1				1	H5		H13
H4	1										1			
H5			1								1	H3	H1	H16
H6	7	5	2	5	3	2	4			12	40			H17
H7						1					1			
H8							1				1			
H9							1				1			
H10							4				4			
H11	3	2		5				2			12	H6		H7
H12	1										1			
H13		1									1			

Total	19	11	10	10	12	14	11	12	12	12	123			

1 = haplotypes from Mexico (Gorrochotegui-Escalante *et al.*, 2002); 2 = haplotypes from Peru (Costa-da-Silva *et al.*, 2005); 3 = haplotypes from the Americas-Africa-Asia (Bracco *et al.*, 2007).

**Table 3:** Genetic variability and neutrality tests of the ten *Aedes aegypti* samples from the Brazilian Amazon.

Populations	NV	*K*	*h*	π	Tajima's *D* test	Fu's *F*s test
Cor	13	5.192	0.737 ± 0.062	0.0136 ± 0.0022	1.456	3.996
Pra	13	5.891	0.745 ± 0.098	0.0155 ± 0.0030	1.432	3.923
Com	5	1.955	0.511 ± 0.164	0.0051 ± 0.0015	0.427	1.926
TaN	12	6.666	0.556 ± 0.075	0.0175 ± 0.0023	2.581	9.078
Coa	4	1.712	0.530 ± 0.136	0.0045 ± 0.0013	1.029	1.900
Stm	9	1.868	0.385 ± 0.149	0.0049 ± 0.0021	-1.304	2.386
Blm	12	6.472	0.782 ± 0.093	0.0170 ± 0.0020	2.518	2.672
Bov	10	3.030	0.303 ± 0.147	0.0079 ± 0.0038	-0.349	5.937
Pac	0	0.000	0.000 ± 0.000	0.0000 ± 0.0000	NC	NC
RBr	0	0.000	0.000 ± 0.000	0.0000 ± 0.0000	NC	NC
Mean	16	4.399	0.666 ± 0.029	0.0115 ± 0.0010	1.309	1.418

NV = number of variable sites; *K* = average number of nucleotide differences; *h* = haplotype diversity; π = nucleotide diversity; NC = not calculated. Significance level for neutrality tests: p > 0.05.

**Table 4:** Genetic distances (*F*_ST_ values) and effective number of migrants (*Nm*), above and below the diagonal, respectively, among samples of *Aedes aegypti* from the Brazilian Amazon.

Population	Cor	Pra	Com	TaN	Coa	Stm	Blm	BoV	Pac	RBr
Cor	-	-0.061	0.055	0.085	0.073	0.084	0.147	0.066	0.228	0.330*
Pra	Inf. (5)	-	0.120	0.000	0.146	0.166	0.087	0.127	0.353*	0.325
Com	8.5 (8)	3.7 (4)	-	0.366	-0.089	-0.037	0.373	0.094	0.214	0.699*
TaN	5.4 (5)	Inf. (10)	0.9 (13)	-	0.398	0.403	0.037	0.302	0.554*	0.476
Coa	6.4 (368)	2.9 (363)	Inf. (360)	0.8 (373)	-	-0.009	0.405	0.111	0.210	0.706*
Stm	5.5 (589)	2.5 (593)	Inf. (597)	0.7 (585)	Inf. (953)	-	0.402*	0.014	0.051	0.732*
Blm	2.9 (1288)	5.3 (1293)	0.8 (1297)	13.1 (1284)	0.7 (1654)	0.7 (702)	-	0.323	0.545*	0.506
BoV	7.0 (656)	3.4 (660)	4.8 (658)	1.2 (654)	4.0 (812)	35.1 (880)	1.0 (1435)	-	0.010	0.716
Pac	1.7 (840)	0.9 (843)	1.8 (841)	0.4 (837)	1.9 (967)	9.3 (1040)	0.4 (1550)	5.0 (185)	-	1.000*
RBr	1.0 (1153)	1.0 (1148)	0.2 (1146)	0.6 (1158)	0.2 (827)	0.2 (1670)	0.5 (2339)	0.2 (1618)	0.0 (1756)	-

*p < 0.001, after Bonferroni correction; Inf. = infinity; geographic distances (km) are in parentheses.
